# Integrated and interprofessional care

**DOI:** 10.5334/ijic.987

**Published:** 2012-08-30

**Authors:** Hugh Barr

**Affiliations:** The Journal of Interprofessional Care, Emeritus Professor of Interprofessional Education, The University of Westminster UK

**Keywords:** integrated care, interprofessional care, interprofessional education

## Abstract

No wonder two movements described in such similar terms are so often confused. One strives to knit services together, the other to cultivate collaborative practice amongst their workers. Dedicated though both of them are to the improvement of health and social care, integrated care falters without engaging the workforce actively as partners in change whilst interprofessional care falters without organisational support. Neither stands alone. Each depends on the other.

## Searching for solutions

Policy makers turn first to organisational ‘solutions’ when services are found wanting—joint planning, joint finance, coordinating machinery and service integration. Outcomes too often disappoint. The integration of some services distances them from others [1], adding to the pressures on staff to collaborate across organisational divides. Implications for the workforce are easily overlooked as reorganisation destabilises working relationships, boundaries are redrawn, power redistributed, roles redefined and services downsized. Recurrent reorganisation demoralises and debilitates, generating stress and prompting defensive behaviour between professions and between organisations [2], at the very time when collaboration may be most critical to implement change in a spirit of give and take. Efforts are redoubled as one round of reorganisation follows another in the confident (or overconfident) expectation that this time the projected improvements will result [3]. The structural fallacy has long since been exposed where policymakers rely on organisational solutions without heeding the human factor [4].

Lessons have been learned the hard way; policy makers today appreciate better than did their forebears the need not only to take workforce planning into account but also education. They call for core curricula across pre-licensure programmes to instill common values, knowledge and skills in the expectation that barriers will then be transcended between the professions freeing up the deployment of personnel in response to the exigencies of the services and the aspirations of the workers. Most recently, the global commission on health professions’ education [5] was noteworthy for embracing both the workforce and the collaborative practice agenda, but stopped short from drawing on IPE sources and resources [6].

## Countering professional resistance

Proposals to liberate roles, responsibilities, and to further career progression, may be welcomed by some professions but resisted by others intent on protecting their territory. They may be united in fearing that the imposition of common curricula will threaten their identities, devalue their distinctive expertise, erode their specialist studies and weaken their control over their education and practice. Resistance, which may be rooted in conservatism and professional self-interest, is exacerbated when educational proposals are presented insensitively with undertones of anti-professionalism. Educational engineering is counterproductive when it becomes a lightning conductor for dissent.

The professions seek reassurance that the distinctive contribution which each makes to practice will be respected and their voice heard when formulating and implementing education and service reforms. Core curricula are incorporated more readily when teachers are accorded time and space to identify commonalities in values, competence and context across professional demarcation as they design and develop the IPE and come to entrust their students with each other. Approached thus, IPE leads into common learning, organically and gradually by mutual consent within acknowledged constraints, reconciling objectives and content for workforce development and collaborative practice operationally and conceptually [7].

## Introducing interprofessional education

But common studies are not enough. They may help in generating a more flexible workforce, but do little, unless and until the experience of the interprofessional movement is taken into account, to further collaborative practice. Including IPE enables the professions to learn with, from and about each other [8]. Then, and only then, may they develop critical appreciation of what each other contribute to collaborative practice in response the increasingly compound and complex needs presented by individuals, families and communities [9].

IPE was grafted on to common curricula in Norway [10] and has coexisted uneasily with such curricula in the United Kingdom since the turn of the century [11]. It was introduced less ambiguously in countries like Sweden and the United States where the lead came from interprofessional activists with governments less involved and, more recently, in those like Australia, Canada and Japan where the implementation of government-led policies has been informed by an interprofessional ethos which is now well documented and readily accessible. Principles have been enunciated [12], competency-based outcomes framed [13–15], theoretical perspectives compared [Barr, H. (forthcoming) towards a theoretical framework for interprofessional education. Journal of Interprofessional Care] and evidence assembled [16, 17].

## Weighing the evidence

Findings from those reviews, corroborated by those from more recent evaluations, confirm that pre-licensure IPE can meet intermediate objectives, namely the modification of reciprocal attitudes and perceptions and the acquisition of shared knowledge bases whereas post-licensure IPE can change practice. Seemingly modest outcomes at the pre-licensure stage have been cited by critics as evidence of the limited impact of IPE, making neither allowance for the immaturity of the students still mastering their respective ‘trades’ nor the competing claims of profession-specific studies on their time, or for the methodological constraints in identifying and controlling intervening variables between the learning and the practice. A more constructive response uses those outcomes as the base baseline for continuing interprofessional development (CIPD) impacting more immediately on practice.

Progress is nevertheless being made in extending and improving outcomes from pre-licensure IPE by introducing more rigorous learning methods like virtual learning environments [18] and laboratory-based simulation to improve hands-on teamwork and patient safety [19], developing team-based interprofessional practice learning [20] and preparing the teachers [21].

CIPD impacts most immediately on service improvement and quality of care when it is employment-based between experienced participants in the same workplace employing methods, such as continuous quality improvement [22], cooperative inquiry [23] and practice professional development planning [24, 25], complemented by university-based post-qualifying programmes to generate a cadre of leaders and to promote progressive models of care. Methods like problem-based learning [26] and, by way of contrast, appreciative inquiry [27], can be applied more readily in pre-licensure IPE to heighten motivation and enhance skills in effecting change.

## Building bridges

I have sought, within the constraints of one short paper, to convey the potential which is being activated in and through work-based interprofessional learning to engage practising professionals positively and constructively as partners in implementing, shaping and sometimes originating change as they harness the power of collaborative interprofessional endeavour. Eschewing arguments that the only effective IPE is in the workplace between experienced practitioners, I have commended pre-licensure IPE as the means to instil the habit of collaborative learning and practice grounded in shared knowledge and the reinforcement of collaborative competence as the growing evidence base confirms.

Much remains to be done to bridge the gap between the formulation of policy and its implementation, to empower practitioners to influence and instigate change, and to open dialogue between policy makers and practitioners. One way is to involve policy and service managers as participants in IPE with practising professionals. Another is to encourage conversation between exponents of integrated and interprofessional care, which is why I welcome the invitation from your editor to highlight some of the issues.

## About the author

Hugh Barr is President of the UK Centre for the Advancement of Interprofessional Education (CAIPE), Emeritus Editor for the Journal of Interprofessional Care, Emeritus Professor of Interprofessional Education and Honorary Fellow at the University of Westminster with visiting chairs at Curtin in Western Australia and Greenwich, Kingston with St George’s London and Suffolk in the UK. He served on the WHO study group on interprofessional education and collaborative practice. His background is in probation, prison aftercare, criminology and social work education.

## References

[1] Leutz W, Glasby J, Dickinson H (2009). Partnership working: key concepts and approaches. International perspectives on health and social care: partnership working in action.

[2] Hinshelwood RD, Skogstad W (2000). Observing organisations: anxiety, defence and culture in health care.

[3] Barr H, Gray R, Walsh K (2012). Interprofessional Education: learning together in health and social care. The Oxford textbook of medical education.

[4] Carrier J, Kendall I, Owens P, Carrier J, Horder J (1995). Professionalism and interprofessionalism in health and community care: Some theoretical issues. Interprofessional issues in community and health care.

[5] Frenk J, Chen L, Bhutta ZA, Cohen J, Crisp N, Evans E (2010). Health professionals for a new century: transforming education to strengthen health systems in an interdependent world. A Global Independent Commission. The Lancet.

[6] Barr H (2011). Engaging with the global challenge. Guest editorial. Journal of Interprofessional Care.

[7] WHO (2010). Framework for action on interprofessional education & collaborative practice.

[8] CAIPE (2008). Interprofessional education—A definition.

[9] WHO (2008). The global burden of disease: 2004 update.

[10] Norwegian Official Report (1986). Coordination in health and social care.

[11] Department of Health (2004). The NHS improvement plan: Putting people at the heart of public services.

[12] Barr H, Low H (2012). Interprofessional education in pre-registration courses: a CAIPE guide for commissioners and regulators of education.

[13] Canadian Interprofessional Health Collaborative (2010). A national competency framework for interprofessional collaboration.

[14] Combined Universities Interprofessional Learning Unit (2010). Interprofessional Capability Framework 2010 Mini-Guide.

[15] Interprofessional Education Collaborative Expert Panel (2011). Core competencies for interprofessional collaborative practice: report of an expert panel.

[16] Barr H, Koppel I, Reeves S, Hammick M, Freeth D (2005). Effective interprofessional education: argument, assumption and evidence.

[17] Hammick M, Freeth D, Reeves S, Koppel I, Barr H (2007). A Best Evidence Systematic Review of Interprofessional Education. Dundee: Best Evidence Medical Education Guide no. 9. Medical Teacher.

[18] Walsh M, van Soeren M (2012). Interprofessional learning and virtual communities: an opportunity for the future. Journal of Interprofessional Care.

[19] Mikkelsen Kyrkjebo J, Brattebo G, Smith-Strom H (2006). Improving patient safety by using interprofessional training in health professional education. Journal of Interprofessional Care.

[20] Barr H, Brewer M, Higgs J, Barnett R, Billett S, Hutchings M, Trede F (2012). Interprofessional practice based education.

[21] Howkins E, Bray J (2008). Preparing for interprofessional teaching: theory and practice.

[22] Walton M (1988). The Deming management method. New York: Putnam Publishing Group WHO (1988) Learning together to work together for health.

[23] Heron J, Reason P, Reason P, Bradbury H (2008). Extending epistemology in cooperative inquiry. Handbook of action research (second edition).

[24] Department of Health (1998). A review of continuing professional development in general practice: a report of the Chief Medical Officer.

[25] Wilcock P, Campion-Smith C, Elston S (2003). Practice professional development planning.

[26] Barrows HS, Wilkerson L, Gijselaers WH (1996). Problem-based learning in medicine and beyond: a brief overview. New directions for teaching and learning.

[27] Cooperrider DL, Whitney D (2005). Appreciative inquiry: a positive revolution in change.

